# Advanced single-strut modelling of lightweight in-filled RC frames: validation and seismic performance assessment

**DOI:** 10.1038/s41598-025-34546-7

**Published:** 2026-01-27

**Authors:** Gehad Abou El-fotuh, Walid A. Attia, M. S. El-Feky

**Affiliations:** 1 Department of Structural Engineering, Faculty of Engineering, Cairo University, Giza, Egypt; 2 Department of Civil Engineering, The Higher Institute of Engineering and Technology Fifth Settlement, New Cairo, Egypt; 3 Department of Civil Engineering, National Research Centre, Giza, Egypt; 4 Heliopolis University for Sustainable Development,, Cairo, Egypt

**Keywords:** Lightweight masonry infills, Finite element modeling, Macro-Model calibration, Seismic performance, Ductility, Parametric analysis, Engineering, Materials science

## Abstract

Accurate numerical simulation of masonry-infilled reinforced concrete (RC) frames is essential for seismic performance assessment, particularly with modern lightweight infill systems like hollow clay bricks (HCB), gypsum blocks (GB), and autoclaved lightweight concrete (ALC) panels. This study presents an advanced single-strut macro-modeling framework, implemented in SAP2000, designed to overcome the limitations of conventional approaches in capturing the distinct nonlinear behavior of these systems. The core innovation involves the development of material-specific nonlinear axial hinges, meticulously calibrated against the full-scale experimental dataset from Cai et al. (J. Earthq. Eng. 23(9):1531-1559, 2019) to replicate crushing (HCB), shear-sliding (GB), and slip-hardening (ALC) mechanisms. The calibrated model demonstrates high predictive accuracy, with errors in lateral capacity and initial stiffness below 10% for all systems. Leveraging this validated model, a comprehensive comparative seismic performance analysis was conducted. The results quantify a critical performance trade-off: HCB infills increased initial stiffness by 471% but exhibited brittle failure, while ALC panels provided superior ductility (µ = 5.39) and stable energy dissipation up to 4.06% drift. The gypsum system confirmed its seismic inadequacy due to abrupt strength degradation. The study concludes that the proposed modeling strategy provides a reliable tool for performance-based design and analysis, enabling engineers to efficiently conduct parametric studies and evaluate the seismic viability of different infill systems at a fraction of the cost and time of full-scale testing.

## Introduction

The seismic performance of reinforced concrete (RC) frame structures is profoundly influenced by the presence of masonry infill walls, a fact tragically underscored by the poor performance of traditional heavy infills during recent seismic events. This has catalyzed a paradigm shift within the structural engineering community towards the utilization of lightweight infill materials, such as hollow clay bricks (HCB), gypsum blocks (GB), and autoclaved lightweight concrete (ALC) panels, which offer a compelling combination of reduced seismic mass and enhanced functional properties. The in-plane behavior of hollow clay brick (HCB) infills has garnered significant research attention due to their complex failure mechanisms under seismic loading. Detailed experimental and analytical assessments have revealed the pronounced anisotropic nature of HCB masonry, primarily attributed to its extrusion-induced material microstructure and perforation geometry^1,2^. Studies quantify the critical influence of mortar joint tensile strength and brick-unit geometry (e.g., horizontal vs. vertical perforations, web thickness) on compressive strut formation and brittle compressive crushing failures under cyclic loads^1–3^. These failures are characterized by low ductility and rapid strength degradation, with drift capacities often below 1.5% for severe damage states^2,3^. Conversely, gypsum block masonry systems are primarily valued for their very low density (400–700 kg/m³ for hollow blocks) and expedient construction, facilitating rapid dry assembly with minimal technical oversight. However, their pronounced structural brittleness and poor tensile strength present a major impediment to seismic application. Under lateral loading, gypsum block infills in reinforced concrete (RC) frames exhibit a shear-sliding failure mode characterized by rapid loss of cohesion at bed joints, leading to abrupt strength degradation at minimal inter-story drift ratios (often below 1.6%), thus offering negligible warning before collapse^4^. This brittle behavior is exacerbated by weak gypsum-mortar substrate bonds and insufficient energy dissipation capacity, as validated experimentally under cyclic conditions. The seminal work of^4^ demonstrated that conventional gypsum block infills undergo diagonal cracking and corner crushing under bidirectional excitation, with stiffness degradation occurring even at low drift demands^4^.In stark contrast, autoclaved lightweight concrete (ALC) panels have emerged as a high-performance lightweight infill system engineered for enhanced seismic resilience. Extensive experimental research, including full-scale cyclic tests, has demonstrated their superior structural behavior, characterized by stable hysteresis loops, significant slip-hardening response, and substantial deformation capacity attributes that collectively improve overall structural ductility and markedly reduce residual drifts. This reduction is critical for post-event reparability and functional recovery, key objectives of modern performance-based seismic design^5,6^. The integration of ALC panels is further justified by a well-documented 40–60% reduction in seismic mass compared to conventional masonry infills, which directly attenuates inertial forces during seismic events. Concurrent benefits include improved thermal and acoustic insulation properties, supporting multi-objective design strategies^5,7^.

Numerical modeling remains an essential methodology for simulating the lateral response of masonry-infilled reinforced concrete (RC) frames, with macro-modeling approaches such as the equivalent diagonal strut method being widely adopted in engineering practice, particularly within software environments like SAP2000. While fiber-based distributed plasticity models are rigorously developed to capture the nonlinear flexural behavior of RC frame elements through explicit integration of material constitutive laws, the representation of masonry infills introduces significant uncertainties. Conventional single-strut macro-models often inadequately represent the complex frame-infill interaction mechanisms, with studies indicating that simplified analytical approaches can overestimate interaction stiffness by 20–35% due to scaling discrepancies and inadequate consideration of strut width formulations^8,9^. Moreover, generalized modeling strategies, including those prescribed by FEMA guidelines, frequently fail to represent the post-cracking behavior and failure modes specific to varied infill material types, particularly lightweight masonry systems. This often leads to inaccurate predictions of strength degradation, hysteretic energy dissipation, and ultimate drift capacity, compromising the reliability of seismic performance assessments^10^.

This study aims to develop a refined and calibrated single-strut macro-model for the nonlinear analysis of RC frames infilled with diverse lightweight masonry systems. The primary objectives are:

1. To formulate material-specific nonlinear hinge properties that accurately capture the unique failure mechanisms of hollow clay brick, gypsum block, and autoclaved lightweight concrete infills.
2. To rigorously calibrate and validate the proposed model against the existing experimental benchmark provided by Cai et al.^11^.
3. To utilize the validated model as a computational tool to conduct a detailed comparative analysis of key seismic performance metrics (stiffness, strength, ductility, energy dissipation) across the different infill systems, extracting insights that support performance-based seismic design.

## Reference test specimens

### Specimen design

**Fig. 1:**
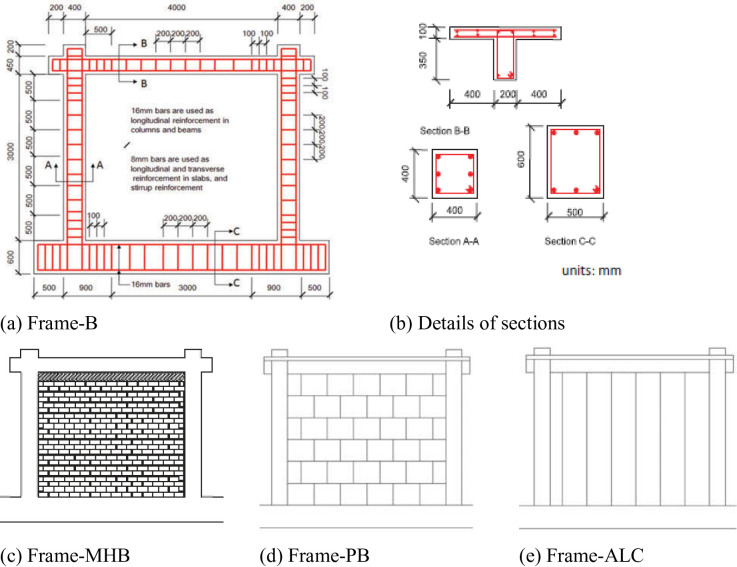
Specimen configurations: (**a**) bare frame (Frame-B); (**b**) cross-section details of beams and columns; (**c**) hollow clay brick infilled frame (MHB); (**d**) gypsum block infilled frame (PB); (**e**) ALC panel infilled frame (ALC). All dimensions in mm.”^11^. .

The experimental investigation evaluated the seismic performance of full-scale reinforced concrete frames with lightweight masonry infills under combined constant axial and reversed cyclic lateral loading^11^. The test matrix comprised four specimens: one bare RC frame (Frame-B) serving as a control and three infilled configurations (Frame-MHB, Frame-PB, Frame-ALC) designed for direct comparison as shown in Fig. 1. All specimens were designed according to Chinese seismic code GB50011-2010 with identical geometry measuring 3450 mm in height and 4000 mm in width. The 400 by 400 mm columns contained six 16 mm diameter longitudinal rebars with 8 mm diameter transverse ties spaced at 100 to 200 mm intervals, while the T-section beams measuring 200 by 450 mm used four 16 mm diameter rebars. A 100 mm thick reinforced concrete slab with a 1000 mm width and minimum volume reinforcements was constructed above the upper RC frame beam in each of the specimens^11^, and strengthened 500 by 600 mm base beam enhanced structural realism as illustrated in Fig. 1.

The MHB and gypsum block infills used identical cement mortar (7–10 mm thick), with MHB frames incorporating five 8 mm connection rebars (700 mm long, spaced at 500 mm) between infill and columns. The ALC panel frame employed slab-to-beam steel connections instead as illustrated in Table 1.

**Table 1:** Specimen matrix.

Specimen	Infill type	Thickness t_mas_ (mm)	Connection details
Frame-MHB	Hollow clay brick	110	8 mm anchors @500 mm
Frame-PB	Gypsum block	200	Mortar bonding
Frame-ALC	ALC panel	100	Slab-to-beam steel connections

Notes: All frames had identical RC members (400 × 400 mm columns, 450 × 200 mm beams) with 6φ16mm column bars and 4φ16mm beam bars.

### Material properties

All materials conformed to relevant Chinese standards including JC/T 698–2010 for plaster blocks and GB 15,762 − 2008 for lightweight concrete panels with complete material properties detailed in Table 2. Material characterization revealed the concrete had an average compressive strength of 33.5 MPa with a modulus of elasticity of 30,000 MPa. The connecting mortar exhibited 5.62 MPa compressive strength and 0.45 MPa tensile strength. The masonry hollow bricks demonstrated anisotropic behavior with 4.2 MPa compressive strengths. Gypsum blocks showed lower strength characteristics at 2.0 MPa compressive strengths, with a density range of 555–563 kg/m³. The autoclaved lightweight concrete panels presented higher performance with 5 MPa compressive strength, 17,500 MPa elastic modulus, and density of 950–960 kg/m³.

### Test setup

The test setup involved anchoring the frame base to a strong floor and applying axial loads (11% of column capacity) and lateral cyclic displacements. Loading began with force control until 0.25% drift, followed by displacement-controlled increments (0.5% to 3.5% drift) to capture nonlinear behavior.

**Table 2:** Material properties^11^.

Types	Main properties	Units	Measurement
Concrete	Compressive strength	MPa	33.5
	Elasticity modulus	MPa	30,000
Steel Reinforcement	Longitudinal, in columns or beam		
	Diameter	mm	16
	Yielding strength	MPa	420
	Transverse, in columns or beam		
	Diameter	mm	8
	Yielding strength	MPa	480
Hollow clay brick	Compressive strength	MPa	4.2
	Density	kg/m³	1800
Gypsum block	Compressive strength	MPa	2.0
	Density	kg/m³	560
	Elasticity modulus	MPa	6500
ALC panel	Compressive strength	MPa	5.0
	Density	kg/m³	955
	Elasticity modulus	MPa	17,500

## Numerical modeling methodology in SAP2000

The seismic performance of all test specimens was numerically investigated using SAP2000 v14 nonlinear analysis capabilities. The modeling approach was carefully calibrated to replicate experimental observations of both bare and infilled RC frames, addressing two primary components: reinforced concrete frame members and masonry infill panels.

### Reinforced concrete frame modeling

**Fig. 2:**
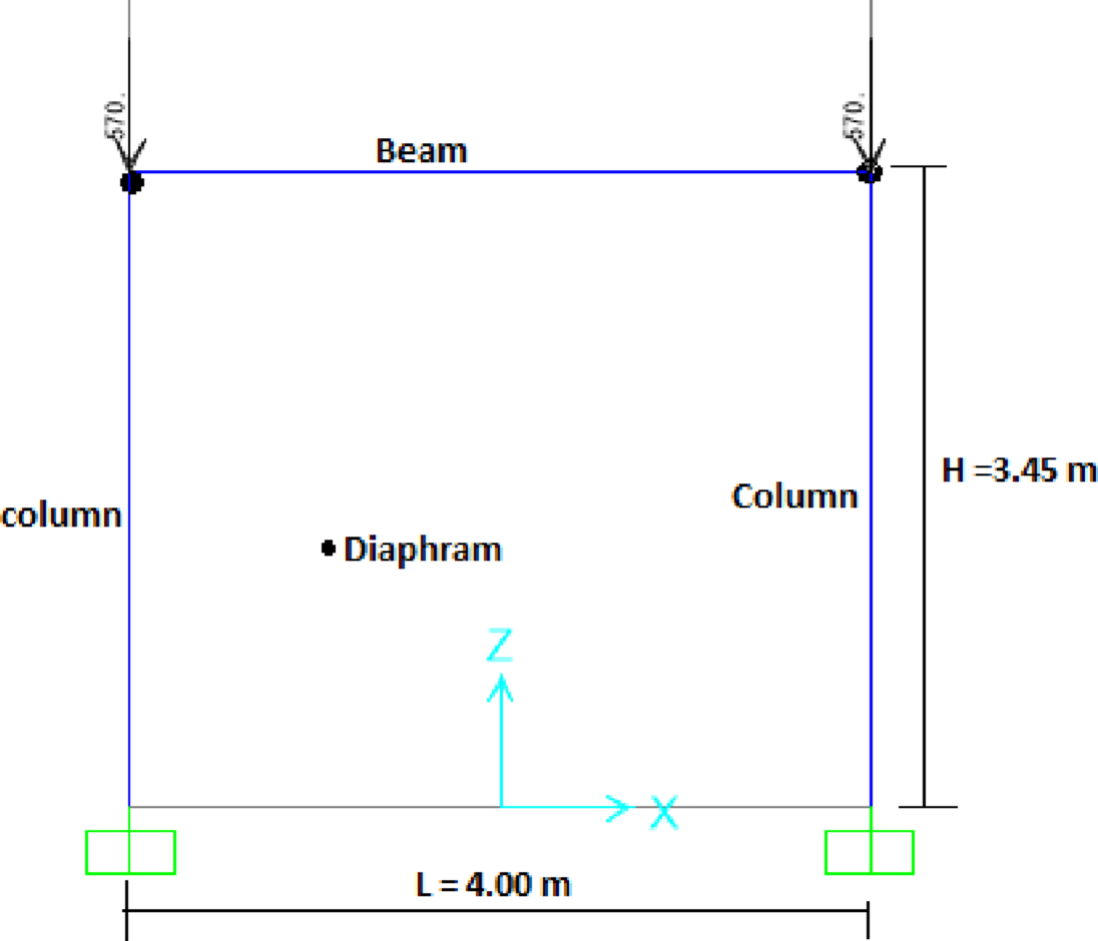
Idealized model of bare RC frame.

The seismic performance of the bare reinforced concrete (RC) frame (Frame-B) was simulated using a **fiber-based distributed plasticity modeling approach**, as illustrated in Fig. 2. This high-fidelity method is recognized for its accuracy in capturing the progressive spread of inelasticity throughout member lengths and across section depths under combined axial-flexural stresses, providing superior predictions of nonlinear cyclic behavior compared to lumped plasticity models^12,13^. The modeling methodology was implemented in three defined steps.

#### Material constitutive modeling

**Fig. 3:**
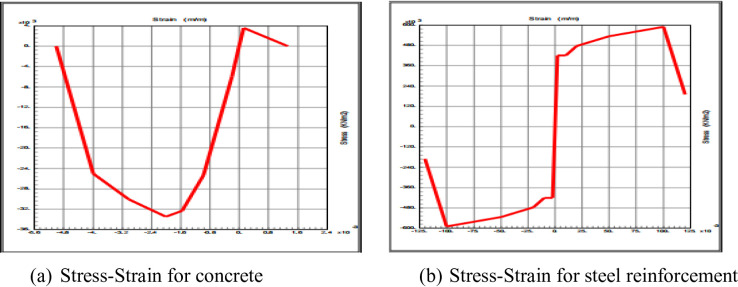
Stress-Strain curve (**a**) concrete (**b**) steel reinforcement.

Material properties were defined in accordance with the Chinese standards (GB50010) referenced in the experimental program. The unconfined concrete (C30) was modeled using the modified Monder model constitutive relationship with a specified compressive strength of 33.5 MPa and a maximum compressive strain (εcu) of 0.003 as shown in Fig. 3. The reinforcing steel (HRB400) was modeled with a kinematic elastoplastic stress-strain curve incorporating strain hardening, using specified yield strengths of 420 MPa for longitudinal bars and 480 MPa for transverse ties as shown in Fig. 3.

#### Fiber-based cross-section definition

**Fig. 4:**
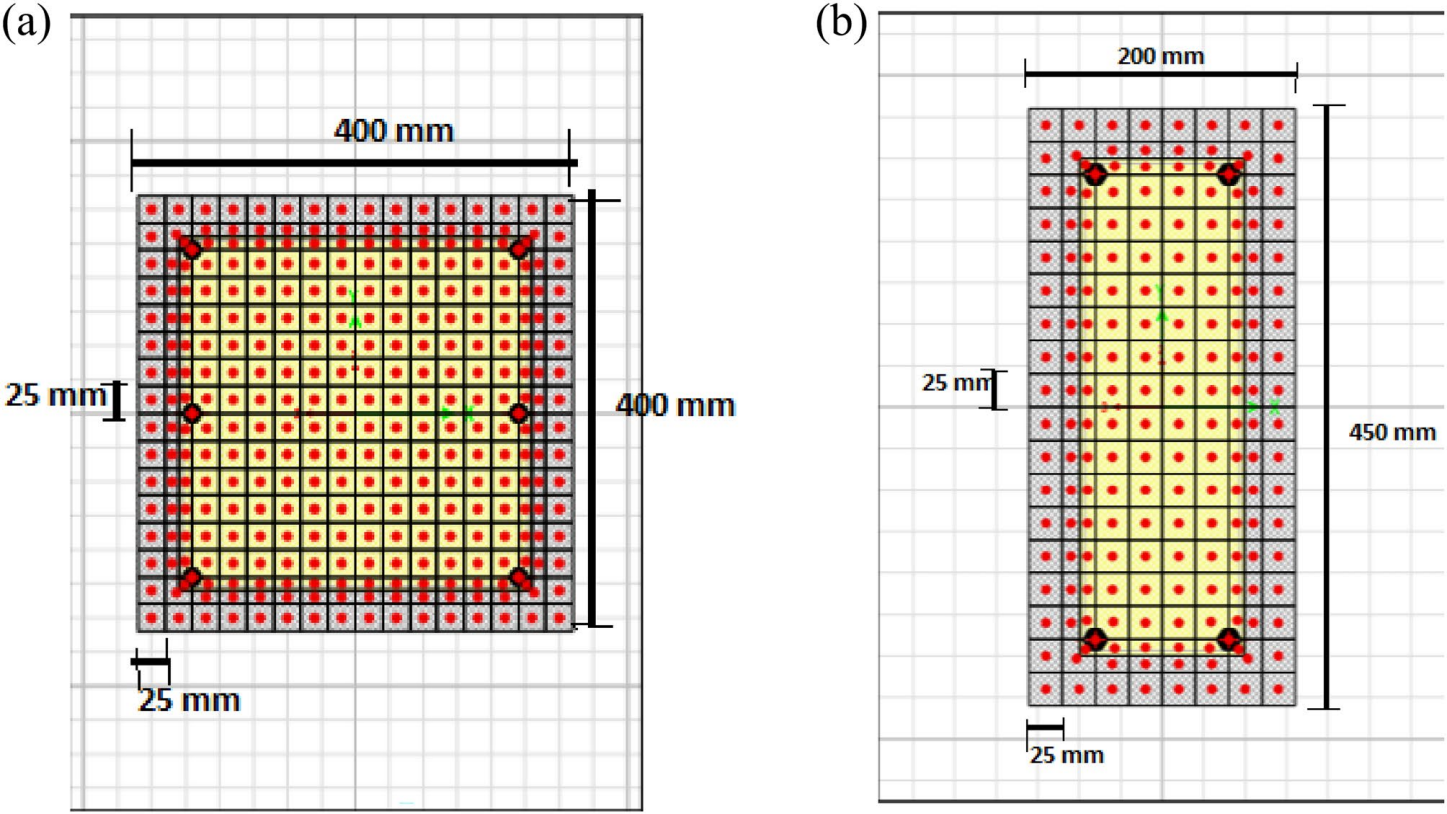
Cross section of (**a**) column and (**b**) beam generated using Section Designer of SAP2000.

The cross-sections for all beams and columns were explicitly modeled in SAP2000’s Section Designer module^14^, precisely replicating the geometric dimensions and the actual reinforcement layout shown in Fig. 4. A consistent 40 mm concrete cover for all members was strictly maintained.

To capture the reduced stiffness of the RC members prior to yielding in the global elastic analysis, a cracked section property modifier was applied. The moment of inertia was set to 0.4*Ig (where Ig is the gross moment of inertia), a well-justified estimate recommended for nonlinear static analysis of RC elements^13^.

For the nonlinear distributed plasticity analysis, the fiber-based approach was implemented by discretizing each section into a mesh of fibers, as shown in Fig. 4. The concrete cover and core were modeled with the Mander law, with the core enhanced by the Mander et al.^15^ confinement model. Reinforcement was explicitly modeled as discrete steel fibers. A refined mesh was employed to ensure convergence and accuracy: For beam and column sections, a maximum fiber size of 25 mm along the flexural axis and perpendicular to it was used.

The nonlinear hysteretic behavior was simulated using a **Fiber P-M2-M3 hinge** type assigned along the length of each frame element. This hinge type automatically utilizes the fiber-discretized cross-section from Section Designer to compute the coupled axial-flexural inelastic response directly from the material constitutive laws at each integration point. The hinges were distributed at multiple locations along the member length to accurately capture the progressive spread of inelasticity throughout the element under combined axial-flexural stresses, providing a robust method for calculating the nonlinear moment-curvature response under varying axial load and superior predictions of cyclic behavior compared to lumped plasticity models^12,13^.

#### Load application and boundary conditions

The numerical model was designed to replicate the experimental loading conditions precisely, as shown in Fig. 2. The base of the columns was modeled with fixed supports, as shown in Fig. 2. To simulate the monolithic action of the concrete slab, rigid diaphragm constraints were applied at the beam level. ensuring realistic lateral force distribution and preventing artificial stress concentrations^16^. A constant axial load of 570 kN was applied as a dead load at the top of each column to simulate sustained gravity loads, as shown in Fig. 2. The lateral loading protocol was simulated using a displacement-controlled nonlinear static (pushover) analysis^17^. This method is computationally stable and effectively captures the full force-deformation response, making it a standard technique for seismic performance assessment.

### Masonry infill modeling strategy

The three distinct lightweight infill systems were simulated using an enhanced equivalent diagonal strut macro-model, a practical approach widely adopted for its computational efficiency in capturing the global lateral response of infilled frames^10^. The conventional single-strut model was significantly modified to account for the unique, material-specific nonlinear behaviors and failure mechanisms observed in the experimental tests.

#### Geometric properties and strut configuration

The geometric properties of the equivalent strut were defined based on the infill panel dimensions. The strut was oriented along the diagonal of the infill panel, with its thickness set equal to the actual thickness of the infill masonry.

The effective width (a) of the diagonal strut was calculated using the well-established Mainstone formulation^18^, which has been widely adopted in guidelines such as FEMA 306^19^:

1 $$\:a=0.175*{\left(\lambda\:*{h}_{col}\right)}^{-0.4}*{d}_{inf}\:\:\:\:\:\:\:\:\:$$

where the non-dimensional infill-frame interaction parameter, λ, is given by:

2 $$\:\lambda\:=\:{\left[\frac{{E}_{me}*{r}_{inf}*{sin}2\theta\:}{4*{E}_{fe}*{I}_{col}*{h}_{inf}}\right]}^{\frac{1}{4}}\:\:\:\:\:\:\:\:\:\:$$

and the diagonal length of the infill panel is:

3 $$\:{d}_{inf}=\sqrt{{h}_{inf}^{2}+{L}_{inf}^{2}}\:\:\:\:\:\:\:$$

and.

h_col​_: Column height between beam centerlines.

h_inf​_, L_inf​_: Height and length of the infill panel.

E_fe_​, E_me​_: Expected modulus of elasticity of the frame and infill material, respectively.

I_col​_: Moment of inertia of the column.

θ: Angle whose tangent is the infill height-to-length aspect ratio = tan − 1(h_inf_/Linf).

**Fig. 5:**
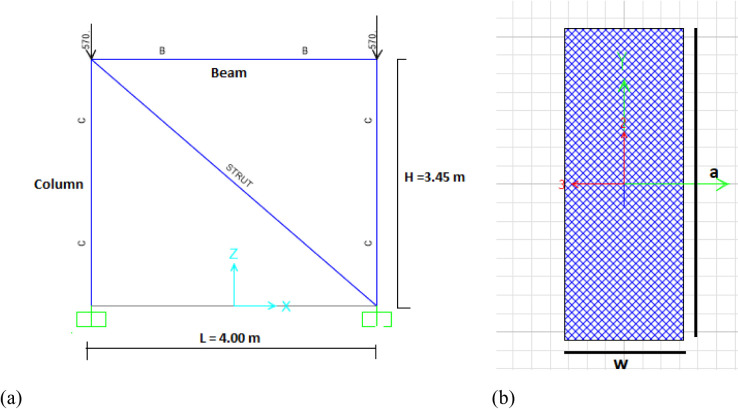
Idealized (**a**) model of masonry infilled RC frame and (**b**) cross-section of strut.

The diagonal strut was connected directly to the beam-column joints of the frame. This connection scheme is representative of the standard equivalent strut macro-model, where the strut idealizes the total diagonal compressive force transferred between the frame and the infill panel. While this approach does not explicitly model the localized contact zones that develop at the corners of the infill under lateral load^20,21^, its global accuracy in capturing the overall lateral strength and stiffness of infilled frames is well-established^10,19^. The idealized model is illustrated in Fig. 5.

**Fig. 6:**
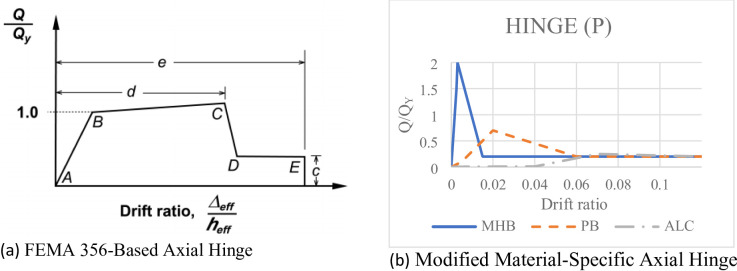
Backbone curves for the axial hinge models: (**a**) Generalized FEMA 356-based hinge; (**b**) Modified Material-Specific Axial hinge.

#### Material-specific nonlinear hinge properties

The accurate numerical representation of the post-cracking nonlinear behavior and failure mechanisms of the infills was the most critical aspect of this study. To achieve this, a sequential modeling strategy was employed, progressively enhancing the model’s complexity and accuracy until a satisfactory correlation with experimental results was achieved.

Strategy 1: Compression-Only Strut with Limit.

The initial approach utilized a simple compression-only strut model, where the frame element representing the diagonal strut was assigned a nonlinear “gap” material property in SAP2000, allowing it to carry only axial compression. A strength limit was defined based on the estimated compressive capacity of the infill material. This model was effective in capturing the initial elastic stiffness and peak strength. However, it proved fundamentally inadequate as it could not simulate the post-peak softening behavior (strength degradation) or the residual strength plateau observed in the experiments. Upon reaching its predefined strength limit, the strut would simply fail and carry zero load, resulting in an unrealistic, abrupt drop in the lateral load-drift response that did not match the more gradual degradation seen in the tests, particularly for the hollow brick and ALC systems.

Strategy 2: FEMA 356-Based Axial Hinge.

To address the limitation of the first strategy, the second approach implemented an axial plastic hinge following the generalized force-deformation relationships recommended in FEMA 356^22^ for masonry infills. The generalized force-deformation relationship for this hinge is conceptually illustrated in Fig. 6a. This hinge defines a backbone curve with an initial linear elastic branch, a yield plateau, and a descending (softening) branch, characterized by parameters for strength (a), and deformation at initial cracking (b), peak strength (c), and residual strength (d).

While this was a significant improvement over the compression-only model, the generic “one-size-fits-all” nature of the FEMA 356 backbone curve proved insufficient. It failed to capture the distinct and material-specific failure mechanisms unique to each lightweight infill system:

It could not accurately represent the extremely brittle, sliding-dominated response of the gypsum blocks, which shows a very steep softening slope. It was unable to replicate the slip-hardening characteristics of the ALC panels after the initial slip. The model provided an average but not optimal fit for the hollow brick’s crushing behavior.

Strategy 3: Modified Material-Specific Axial Hinge.

The final and adopted strategy involved the development of customized nonlinear axial hinge properties for each infill material type as shown in Fig. 6b. The backbone curves for these hinges were not based on a generic guideline but were directly calibrated against the experimental force-deformation data obtained from the cyclic tests^11^. This allowed for the precise definition of the post-peak softening slope and residual strength plateau.

The calibrated hinges were designed to capture the following distinct behaviors:

Hollow Clay Brick (MHB): A steep post-peak softening slope (approximately − 0.8P_max/Δ) was defined to model the rapid strength degradation due to brittle compressive crushing of the bricks and mortar. A residual strength plateau of ~ 50% of P_max was set to represent the fractured masonry debris continuing to provide some compressive resistance.

Gypsum Block (PB): The hinge model was calibrated to replicate the shear-sliding failure. It features an abrupt strength drop at cracking (simulating bed joint failure), down to a very low residual frictional plateau (~ 20% of P_max), with no strain hardening, consistent with the material’s brittle nature.

ALC Panel (ALC): The hinge properties were formulated to simulate its unique slip-hardening characteristics. The model captures the initial elastic response, a pronounced yield plateau due to panel slippage, followed by a gradual strain-hardening phase, allowing the strut to maintain a high percentage of its peak capacity up to large drift levels.

The assignment of these calibrated, material-specific hinges was the pivotal factor that enabled the high fidelity of the final model. It successfully captured not only the initial stiffness and peak strength but also the complex post-peak behavior, including the steep softening slopes and residual capacities, as validated in Sect. "Model validation and lateral load-drift response".

This sophisticated numerical approach provided the necessary foundation for reliably evaluating the seismic performance of the lightweight infill systems, enabling direct comparison with experimental results across all response phases.

## Results and discussion

The developed numerical models were rigorously validated against experimental data to evaluate their predictive accuracy for key seismic performance indicators. This section presents a comparative analysis of the lateral load-drift behavior, failure mechanisms, and overall performance metrics (stiffness, ductility, and energy dissipation) for all four frame configurations. The discussion synthesizes these results to provide critical insights into the seismic viability of different lightweight infill systems.

### Model validation and lateral load-drift response

**Fig. 7:**
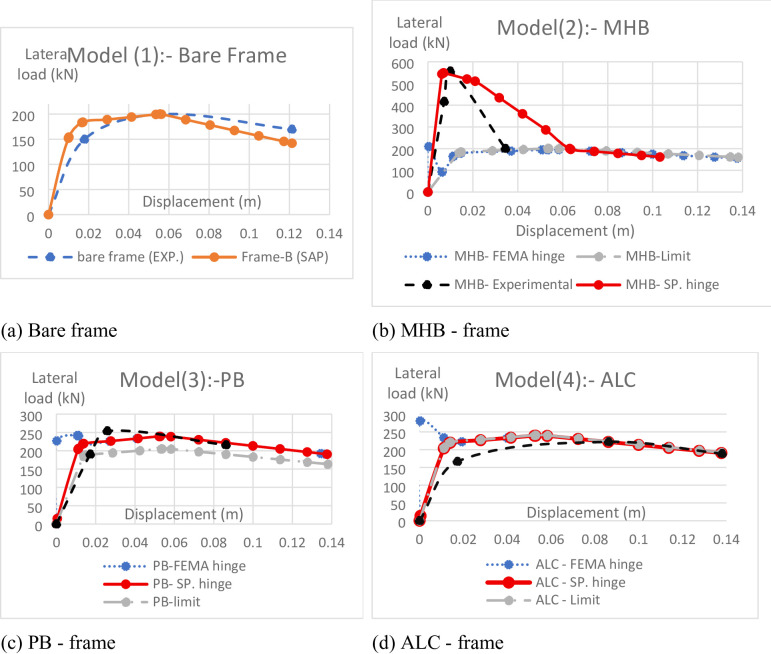
Comparison of lateral behavior in the experiment and numerical model. (**a**) Bare frame, (**b**) MHB - frame, (**c**) PB - frame, (**d**) ALC - frame.

The hysteresis backbone curves derived from the experimental tests and numerical simulations are compared in Fig. 7. The close agreement across all frame types demonstrates the efficacy of the enhanced single-strut modeling approach, particularly the modified material-specific axial plastic hinges.

#### Bare RC frame (Frame-B)

As shown in Fig. 7a, the numerical model of the bare frame accurately replicated the classic elastoplastic response. The simulation predicted the yield drift (0.63% vs. 0.67% experimental) and peak lateral strength with errors of less than 3%. The post-yield strength degradation slope was also captured with high fidelity, confirming the suitability of the fiber-based distributed plasticity model for simulating the flexural yielding of RC members under cyclic loading.

#### Hollow clay brick-infilled frame (Frame-MHB)

The lateral response of the hollow clay brick-infilled frame (Frame-MHB), presented in Fig. 7b, displays a characteristic trilinear response marked by high initial stiffness, a distinct loss of stiffness after diagonal cracking, a peak strength plateau, and a post-peak strength degradation phase. The progression of the numerical modeling strategies from simple to sophisticated demonstrates a clear enhancement in capturing this complex behavior, culminating in the high fidelity of the final, adopted model.

- **Strategy 1: Compression-Only Strut with Limit (curve ‘MHB-limit’)**: This initial, simplistic approach successfully captured the system’s high initial elastic stiffness, as the single strut accurately represents the diagonal compression action of the infill panel. However, its fundamental inadequacy is starkly revealed upon reaching the predefined compressive strength limit. The strut fails abruptly, resulting in a complete and instantaneous loss of load-carrying capacity. This unrealistic behavior contrasts sharply with the experimental curve, which shows a gradual degradation of strength after peak load. This strategy fails entirely to simulate the post-peak residual capacity provided by the fractured masonry debris and frictional mechanisms.
- **Strategy 2: FEMA 356-Based Axial Hinge (curve ‘MHB- FEMA hinge’)**: The implementation of a generalized FEMA 356 backbone curve was a significant improvement. It moved beyond a simple strength limit to model a more realistic post-peak softening branch, preventing the abrupt failure seen in Strategy 1. This model provided a reasonable prediction of the peak strength. However, its “one-size-fits-all” formulation could not accurately replicate the specific degradation slope and residual strength plateau unique to hollow clay brick masonry. The generic hinge over-simplified the complex failure mechanism involving progressive crushing of bricks and mortar, leading to a discrepancy in the softening phase.
- **Strategy 3: Modified Material-Specific Axial Hinge (curve ‘MHB – SP. hinge’)**: The final, adopted strategy involved a custom-calibrated hinge based directly on the experimental data. This model successfully integrates the strengths of the previous strategies while eliminating their weaknesses. It accurately captures the initial elastic stiffness (87.5 kN/mm experimental vs. simulated) and the ~ 30% reduction after diagonal cracking at 0.3% drift. Most importantly, it precisely defines the post-peak behavior: a steep softening slope (approximately − 0.8P_max/Δ) to model the rapid strength degradation from brittle compressive crushing, followed by a residual strength plateau at ~ 50% of P_max to represent the continued frictional and compressive resistance of the cracked infill.

The close alignment of the ‘MHB – SP. hinge’ curve with the experimental curve validates this material-specific approach. The minor conservative estimation of residual capacity (a 12% overestimation at 2% drift) can be attributed to the challenge of perfectly calibrating the crushing strain limits and the evolving friction coefficient in the strut formulation against the complex, progressive damage observed in the physical test. This strategy proved essential for replicating not just the peak strength (1.5% error) but the entire force-deformation history that defines the seismic performance of hollow clay brick infills.

#### Gypsum block-infilled frame (Frame-PB)

The lateral response of the gypsum block-infilled frame (Frame-PB), shown in Fig. 7c, is characterized by its pronounced brittleness, featuring a sudden and significant strength loss immediately after cracking. This abrupt failure mechanism, dominated by shear-sliding at the bed joints, provides a critical test for the three numerical strategies, highlighting the limitations of conventional approaches and the necessity of a highly tailored model.

- **Strategy 1: Compression-Only Strut with Limit (curve ‘PB-limit’)**: This model was fundamentally inadequate for capturing the behavior of the gypsum infill. While it could simulate the initial elastic stiffness with reasonable accuracy, its failure mechanism is entirely misrepresentative. Upon reaching the compressive strength limit, the strut fails completely, unloading abruptly to zero load. This simulation implies a pure crushing failure, which is inconsistent with the experimental curve. The actual failure was a shear-sliding mechanism, where the infill panel lost most of its compressive capacity but retained a low, frictional residual strength. Strategy 1 fails to capture any post-cracking resistance, leading to a gross underestimation of the system’s behavior beyond the peak.
- **Strategy 2: FEMA 356-Based Axial Hinge (curve ‘PB -FEMA hinge’)**: The implementation of the generalized FEMA 356 backbone curve represented a theoretical improvement by introducing a post-peak degradation branch. However, its formulation is too generic to capture the extreme brittleness of gypsum. The standard hinge imposes a gradual softening slope and often a non-zero residual strength plateau that is too high and too elongated. As a result, while Strategy 2 predicts a strength degradation, it does so too gradually. It fails to replicate the dramatic, almost instantaneous **38% strength drop** observed in the test at approximately 0.9% drift, significantly overestimating the force capacity in the immediate post-cracking phase.
- **Strategy 3: Modified Material-Specific Axial Hinge (curve ‘PB – SP. hinge’)**: The final, calibrated strategy was specifically designed to overcome the limitations of the previous models by directly emulating the shear-sliding failure mode. The backbone curve for this hinge was custom-calibrated to feature: (1) an abrupt strength drop at cracking to simulate the immediate loss of cohesion upon bed joint failure, and (2) a very low residual frictional plateau (~ 20% of P_max) with no strain hardening, consistent with the material’s brittle nature and reliance on friction alone after sliding initiates. This strategy successfully predicted the cracking load and the drift level at which the sudden strength loss occurred, with an error of only 5%. The model also captured the low, flat residual strength phase. However, even this advanced approach overestimated the residual strength by **22%**. This persistent discrepancy underscores the immense challenge of numerically capturing the progressive loss of friction and the rapid propagation of microcracking through the weak gypsum material. The complexity of simulating the exact post-cracking interface behavior remains a limitation of the macro-modeling approach for extremely brittle infills.

The evolution of the models for Frame-PB clearly demonstrates that for highly brittle materials like gypsum, neither simple nor generalized modeling strategies are sufficient. While Strategy 3 provided the best correlation and was crucial for capturing the overall brittle nature of the response, it also highlighted the practical limits of the single-strut approach in perfectly replicating the most severe degradation mechanisms.

#### ALC panel-infilled frame (Frame-ALC)

The lateral response of the ALC panel-infilled frame (Frame-ALC), presented in Fig. 7d, is distinguished by its unique slip-hardening behavior, which presents a fundamentally different challenge for numerical modeling compared to the brittle infills. The performance of the three strategies in capturing this behavior characterized by a stable yield plateau and a subsequent strength increase clearly demonstrates the necessity of a specialized modeling approach that can replicate this ductile, stable mechanism.

- **Strategy 1: Compression-Only Strut with Limit (curve ‘ALC-limit’)**: This model was entirely inadequate for simulating the ALC panel’s response. While it could capture the initial elastic stiffness, its fundamental premise leads to a complete misinterpretation of the failure mode. Upon reaching its predefined compressive strength limit, the strut fails abruptly and unloads to zero. This simulation implies a brittle crushing failure, which is entirely inconsistent with the experimental curve. The actual physical behavior is dominated by panel slippage and re-engagement, not crushing. Consequently, Strategy 1 fails to capture the most defining features of the ALC response: the pronounced yield plateau and the subsequent strain-hardening phase, severely underestimating both the deformation capacity and the energy dissipation.
- **Strategy 2: FEMA 356-Based Axial Hinge (curve ‘ALC – FEMA hinge’)**: The use of the generalized FEMA 356 backbone curve offered a marginal improvement by introducing a post-peak degradation branch. However, its standardized formulation is designed for conventional, degrading materials and is incapable of capturing the unique **strain-hardening** exhibited by the ALC panels. The FEMA hinge typically includes a descending (softening) branch after peak strength, which is the opposite of the hardening behavior observed in the test. Therefore, while it may roughly approximate the initial peak, Strategy 2 completely fails to replicate the sustained load capacity and increasing strength at higher drift levels, leading to a significant underestimation of the system’s true performance and ductility.
- **Strategy 3: Modified Material-Specific Axial Hinge (curve ‘ALC – SP. hinge’)**: The final, adopted strategy was specifically engineered to replicate the unique slip-hardening mechanism of the ALC panels. The backbone curve for this hinge was custom-calibrated to include: (1) an initial linear elastic branch, (2) a pronounced yield plateau to accurately represent the slippage of the panel within the frame, and (3) a crucial **gradual strain-hardening branch** to simulate the re-engagement and increased compressive resistance of the panel after initial slip. This strategy achieved exceptional fidelity with the experimental results. It accurately captured the initial stiffness (14.8 kN/mm experimental vs. 14.6 kN/mm simulated) and, most importantly, the entire load plateau between 0.6 and 1.2% drift within a 3% error in both drift magnitude and load level. The model also successfully replicated the subsequent hardening response. The slight overestimation of this hardening phase (by approximately 7% at higher drifts) is a reasoned consequence of the model’s idealized assumptions regarding the perfect re-engagement and interface condition between the panel and the frame after major slippage has occurred.

The progression of models for Frame-ALC underscores a critical finding: for advanced infill systems exhibiting non-conventional, ductile mechanisms like slip-hardening, only a meticulously calibrated, material-specific approach can yield accurate results. Strategy 3 was not merely an improvement but a necessity to move beyond the limitations of conventional models that are inherently designed for strength degradation. Its success validates the proposed enhanced single-strut framework for simulating the next generation of seismic-resistant infill systems.

Summary of Validation: The validated models achieved an average error of less than 10% in predicting initial stiffness and peak strength. The most significant discrepancies occurred in modeling the post-peak behavior of brittle systems (e.g., gypsum), underscoring the necessity of material-specific hinge properties and identifying an area for future model refinement.

### Comparative seismic performance assessment

**Fig. 8:**
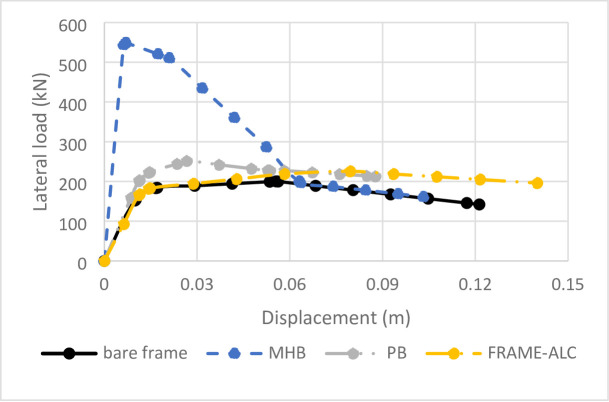
Comparison of lateral behavior of bare frame with different type of infill.

The validated models enable a detailed quantitative comparison of seismic performance metrics, as summarized in Table 3; Fig. 8. The analysis reveals fundamental trade-offs between stiffness, strength, ductility, and energy dissipation.

**Table 3:** Comparative seismic performance metrics for all frame specimens.

Model	Initial stiffness (kN/mm)	Post-cracking stiffness (kN/mm)	Ductility ratio (µ)	Ultimate drift ratio (%)	Total dissipated energy (J)
Bare Frame	15.32	10.76	3.25	3.52	23,364.28
MHB	87.51	19.23	2.47	2.99	35,099.13
PB	18.43	15.15	1.81	2.54	19,288.55
ALC	14.80	12.39	5.39	4.06	28,269.63

Where Post-Cracking Stiffness is secant stiffness at peak strength.

#### Stiffness and strength considerations

The incorporation of infills drastically altered the lateral stiffness of the frames. The hollow brick infill (Frame-MHB) provided the most substantial benefit, increasing initial stiffness by 471% compared to the bare frame (Table 3). This highlights its potential to reduce inter-story drifts under service-level earthquakes. Conversely, the ALC panel (Frame-ALC) offered minimal stiffness enhancement, behaving more like a bare frame in the elastic phase. The gypsum block (Frame-PB) provided a moderate 20% stiffness increase, but this benefit was instantly lost after its brittle failure at low drift levels, as clearly depicted in the stiffness degradation plot of Fig. 8.

#### Ductility and deformation capacity

The ductility ratios (µ), compared in Table 3, reveal a critical inverse relationship between initial stiffness and deformation capacity. The brittle nature of the hollow brick and gypsum infills significantly reduced the system’s ductility (µ = 2.47and 1.81, respectively) compared to the bare frame (µ = 3.25). In stark contrast, the ALC-infilled frame achieved a superior ductility ratio of 5.39, withstanding drifts up to 4.06% before significant strength loss. This exceptional performance is attributed to its ability to undergo controlled slippage and yielding, dissipating energy without brittle fracture.

#### Energy dissipation mechanisms

The total energy dissipation, calculated from the area under the load-drift curves and compared in Fig. 8; Table 3, provides insight into the systems’ seismic resilience.

- Frame-B showed stable energy dissipation (23,364 J) through distributed plastic hinge formation in the RC frame.
- Frame-MHB dissipated the most energy (35,099 J) due to its high initial strength. However, 72% of this occurred in the unstable post-cracking phase, involving non-repairable damage from masonry crushing.
- Frame-PB dissipated the least energy (19,289 J) through undesirable mechanisms like uncontrolled cracking and pinched hysteresis, offering little reliable damping.
- Frame-ALC demonstrated highly efficient energy dissipation (28,270 J). Crucially, 82% of this energy was absorbed through stable, repeatable elastic-plastic cycles (evident in its stable hysteresis loops), indicating that the majority of deformation was repairable, a key advantage for post-earthquake functionality.

### Synthesis and practical implications

The results, synthesized from Figs. 7 and 8; Table 3, challenge the conventional paradigm that equates higher infill stiffness with better seismic performance. Instead, they advocate for a performance-based approach that prioritizes controlled damage and repairability.

1. Hollow Clay Bricks: While beneficial for wind and minor seismic loads, their brittle failure and uncontrolled damage progression (evident in the rapid stiffness degradation in Table 3) pose a significant risk during major earthquakes. Their use in high-seismicity regions requires careful consideration and potentially supplemental strengthening.
2. Gypsum Blocks: Their extremely low ductility (Table 3) and brittle failure mechanism render them unsuitable as structural infills in seismic regions. Their use should be limited to non-structural partitions in zones of low seismicity.
3. ALC Panels: This system emerges as the most promising for seismic applications. By sacrificing initial stiffness for exceptional ductility and stable, repairable hysteresis (Table 3), ALC infills provide a resilient solution that aligns with modern performance-based design objectives. They allow a structure to “ride out” a seismic event through large, controlled deformations without catastrophic collapse.

This study provides engineers with validated numerical tools and quantitative data to make informed decisions, favoring systems like ALC that ensure life safety and enhance post-earthquake recoverability.

## Conclusions

This study has established a computationally efficient and experimentally validated macro-modeling framework for the seismic analysis of RC frames with lightweight masonry infills. The principal contribution is the development and implementation of calibrated, material-specific nonlinear hinges within a single-strut model, effectively simulating the complex in-plane behavior of hollow clay brick, gypsum block, and ALC panel infills.

The model was successfully calibrated against the experimental benchmark from Cai et al.^11^, achieving high accuracy in predicting global response parameters. This validated model served as a foundational tool to execute a systematic comparative performance assessment that extends the findings of the original experimental work. The analysis unequivocally demonstrates that:

- The significant stiffness enhancement (471%) offered by **Hollow Clay Brick** infills is offset by their brittle failure mechanism, posing a significant risk for structural integrity under strong seismic motions.
- **Gypsum Block** infills, characterized by an abrupt shear-sliding failure and minimal energy dissipation, are seismically unsuitable for use as structural elements.
- **ALC Panels** represent the most resilient option, with the model successfully capturing their unique slip-hardening behavior, which leads to exceptional ductility (µ > 5.0) and stable, repairable hysteresis.

The proposed modeling framework provides researchers and practicing engineers with a practical and high-fidelity tool for the performance-based seismic design and assessment of infilled frames. It facilitates a paradigm shift in infill selection from a stiffness-centric approach to one that prioritizes deformation capacity and controlled damage, ultimately enhancing seismic resilience and post-event functionality.

### Limitations and future work

While the model demonstrates high predictive accuracy for the three lightweight infill systems considered, it is important to acknowledge that the calibration and validation were performed against a single comprehensive experimental dataset (Cai et al.^11^). This dataset was selected for its unique inclusion of HCB, GB, and ALC infills under identical frame conditions, providing a robust benchmark for comparative analysis. Future work should extend validation to independent experimental studies with varied frame geometries, infill configurations, and loading protocols to further generalize the proposed modeling framework. Nevertheless, the methodology presented based on material-specific hinge calibration provides a reliable and transferable approach for simulating the distinct failure mechanisms of modern lightweight infills.

## Data Availability

All data generated or analyzed during this study are included in this published article.
